# Black thyroid in a dog on long‐term doxycycline therapy

**DOI:** 10.1111/vsu.70041

**Published:** 2025-10-13

**Authors:** Sarah J. Stark, Alexandra R. Armstrong, Joshua L. Merickel, Wanda J. Gordon‐Evans

**Affiliations:** ^1^ Veterinary Clinical Sciences Department College of Veterinary Medicine, University of Minnesota Saint Paul Minnesota USA

## Abstract

**Objective:**

To increase awareness of black thyroid in dogs and to prevent unnecessary total thyroidectomy. A benign condition called “black thyroid” has been documented in greater than 250 people on chronic minocycline therapy, and rarely in animals. To our knowledge this is the first report of black thyroid in an animal secondary to doxycycline therapy.

**Study design:**

Case report.

**Animal:**

One 10 year‐old female spayed Collie‐cross dog.

**Methods:**

A dog on long‐term doxycycline underwent a right parotid sialoadenectomy and left thyroidectomy to remove associated tumors. Black pigmentation of both thyroid lobes was observed intraoperatively. The left thyroid gland and associated nodule were excised, leaving the right lobe intact.

**Results:**

Histopathology of the left thyroid nodule and right parotid salivary gland were consistent with thyroid follicular‐compact cell carcinoma with metastasis. Finely granular brown pigment was present multifocally within the cytoplasm of many of the thyroid follicular cells and extracellularly within the colloid as irregular gray to brown glassy aggregates. The pigment was negative for iron and calcium and had minimal to no immunoreactivity for melanin.

**Conclusion:**

These findings aligned with those reported for the condition black thyroid in humans. At this time, there is no evidence that performing a thyroidectomy is necessary or appropriate for black thyroid. Veterinary surgeons should be aware that dogs on long‐term doxycycline therapy may have this discoloration, so unnecessary total thyroidectomy can be prevented in affected animals.

## 1 INTRODUCTION

A condition referred to as “black thyroid” has been documented in greater than 250 people on chronic minocycline therapy since the 1970s^1^, ^2^ and even more rarely in those chronically on doxycycline, another second‐generation tetracycline antibiotic.^3^ Minocycline has been demonstrated to produce this black pigment through an oxidation reaction with thyroid peroxidase.^4^, ^5^ The dog described in the present report was prescribed doxycycline for 3 years prior to thyroidectomy, as a component of treatment for discoid lupus erythematosus. While black thyroid has been reported in dogs, rats, and monkeys during initial drug trials for minocycline,^6^ to our knowledge black thyroid in a dog secondary to minocycline administration has only been reported once outside of experimental settings.^7^ In addition, we believe black thyroid has not previously been reported in dogs receiving doxycycline. A search of PubMed and Google Scholar using the keywords “‘black thyroid’ AND doxycycline AND animal,” yielded 45 manuscripts. Many of these cited Benitz, who first reported black thyroid in laboratory animals.^6^ None documented black thyroid in a dog or other animal on doxycycline.

As black thyroid is generally first diagnosed during surgery, its discovery is inherently unexpected and alarming. The goal of this report is to increase awareness of this condition in veterinarians and to prevent unnecessary total thyroidectomy upon the discovery of black thyroid in dogs.

## 2 CASE REPORT

A 10‐year‐old female spayed Collie‐cross dog was presented for a surgical consult for a right parotid salivary gland mass. The dog's only major medical history was a diagnosis of lupus erythematosus, which was managed by a board‐certified veterinary dermatologist for approximately 3 years prior to presentation to our hospital. During this 3‐year period, the dog was prescribed a protocol of oral medications including 1000 mg of fish oil daily, 18–36 mg/kg niacinamide daily, and 5.5–11 mg/kg doxycycline daily. These medications were well tolerated, and the dog's disease was controlled.

**FIGURE 1:**
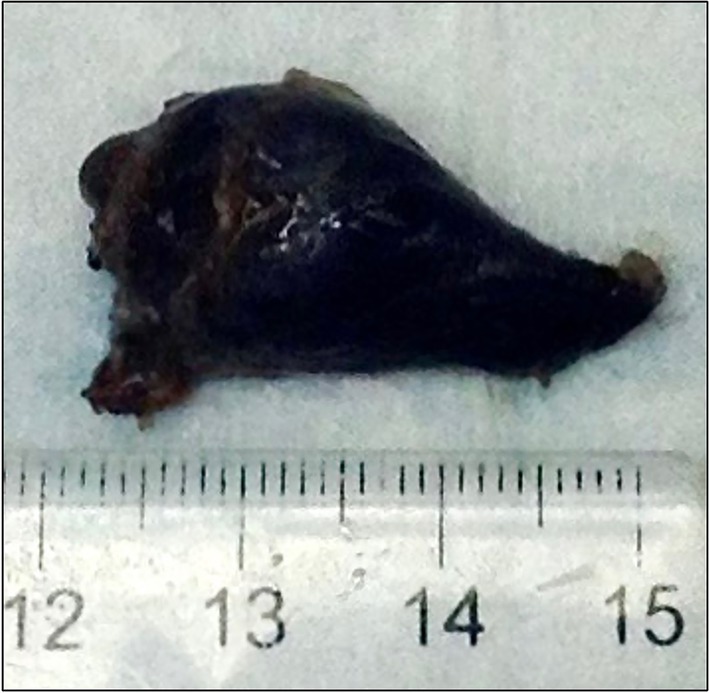
The resected left lobe of the thyroid and associated nodule, which is grossly diffusely black.

After consulting with the surgery service, the owners elected to proceed with computed tomography (CT) of the head, neck, and thorax for surgical planning and staging. Imaging showed a 3.8 × 3.7 × 2.2 cm right parotid salivary gland mass and an additional 2.0 × 1.5 cm left thyroid nodule. There was no evidence of lymphadenopathy or distant metastasis. No aspirate or biopsy of the thyroid nodule was obtained at the time of the CT. Surgery was scheduled to remove both masses 9 days later. A right parotid sialoadenectomy with associated lymph node extirpation was performed using a standard approach. The dog was repositioned to dorsal recumbency and a ventral midline approach to the thyroid gland was performed. Both lobes of the thyroid were exposed and visually inspected. The left and right lobes were diffusely black. The nodule seen on CT was present in the left lobe, and the right lobe was normal in shape, size, and palpation. No adjacent tissues were grossly discolored, and the retropharyngeal lymph nodes were unremarkable. Due to concern for melanocytic neoplasia, an oral examination was performed but no masses were seen. The owner was informed intraoperatively, and elected to proceed with the removal of the left lobe of the thyroid and associated nodule only, leaving the right lobe intact. The left lobe was photographed following resection to show the abnormal color (Figure 1).

**FIGURE 2:**
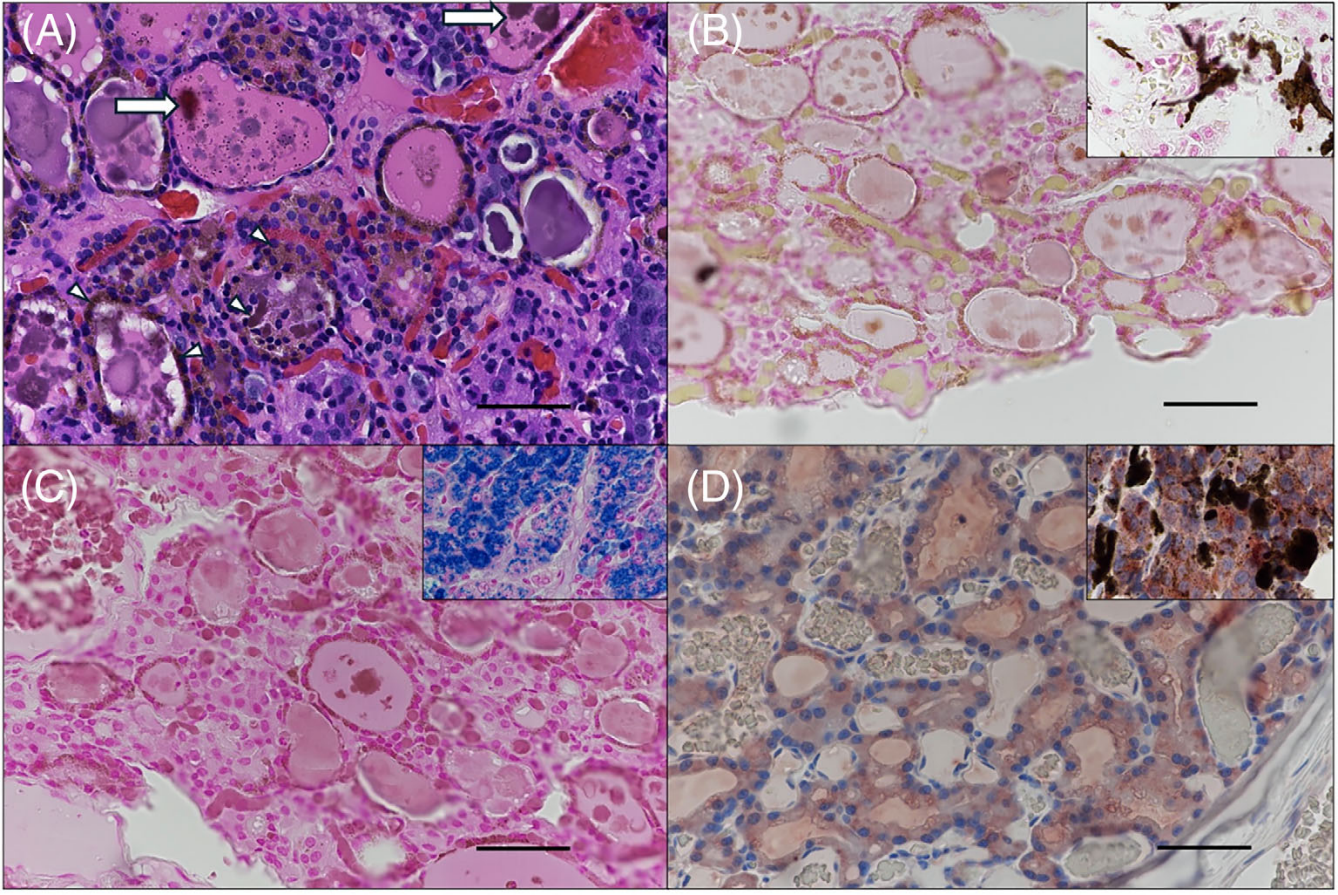
Histologic appearance of affected thyroid gland. (A) Brown pigment was present within the colloid in irregular globules up to 25 μm diameter (arrows) and as fine pinpoint 1–2 μm diameter granules within the epithelial cells (arrowheads). (B) The pigment was negative on a von Kossa stain for calcium and (C) Prussian blue stain for iron and (D) negative on Melan—A immunohistochemistry (IHC) for immunoreactivity. Insets show positive controls for the special stains and IHC. Magnification = 400×; scale bars = 50 μm.

Histopathology of the left thyroid nodule was consistent with a solid (compact) follicular thyroid carcinoma. The mass was relatively well‐demarcated, partially encapsulated, and nodular. It was composed of a proliferation of simple cuboidal to low columnar epithelial cells that were arranged in clusters or formed irregular follicular structures containing minimal colloid. The epithelial cells had moderately sized, round, central euchromatic nuclei with a single, moderately sized nucleolus. Nuclear pleomorphism was minimal and mitotic figures were uncommon. The mass was completely excised with focally narrow tumor‐free margins (91 μm). Multifocally, finely granular brown pigment was present within the cytoplasm of many of the thyroid epithelial cells and extracellularly as irregular gray to brown glassy aggregates up to 25 μm diameter within and surrounded by colloid (Figure 2A,B). Interestingly, pigment was largely confined to the non‐neoplastic thyroid gland adjacent to the neoplasm. To further characterize the pigment, special stains (Prussian blue for iron, von Kossa for calcium) and immunohistochemistry for Melan‐A (melanin) were performed. The pigment was negative for iron and calcium and had minimal to no immunoreactivity for melanin (Figure 2C,D). Sections of lymph node and adjacent salivary glandular tissue were extensively effaced and replaced by a proliferation of simple cuboidal to low columnar epithelial cells arranged in clusters or forming irregular follicular structures containing minimal colloid. The right parotid salivary gland mass and an associated lymph node were determined to be metastatic carcinoma originating from the left thyroid mass. There was no discoloration of this tissue grossly or microscopically.

The dog recovered uneventfully from surgery and was discharged from the hospital with a nonsteroidal anti‐inflammatory drug for analgesia. Monitoring of thyroid function and calcium levels were not performed postoperatively. No postoperative complications were reported. Aside from the initial surgical intervention, no further treatment for the carcinoma was pursued by the owners. The dog was euthanized approximately 6 months after surgery due to collapsing episodes of unknown etiology.

## 3 DISCUSSION

A full thyroidectomy was discussed at the time of surgery due to the abnormal coloring of the thyroid lobes bilaterally. This would have proven to be unnecessary and would have resulted in increased morbidity for the patient and cost to the owner, requiring calcium and thyroid hormone monitoring and supplementation. In cases of black thyroid, discoloration of the thyroid tissue is attributed to minocycline reacting with thyroid peroxidase, which produces the black pigment.^4^ Histopathologic reports in humans detail black to brown granular material in the colloid, lack of iron deposits, fluorescence, and lipofuscin,^5^, ^8^, ^9^ similar to what was found in this case. In cases of black thyroid with concurrent papillary thyroid cancer, a common histologic finding is a lack of black pigmentation in the tumor itself, with pigment being found only in the normal parenchyma, which also aligned with what we observed in this case.^5^, ^9^, ^10^

In humans, minocycline is often prescribed for the treatment of acne vulgaris.^10^, ^11^ The discovery of black thyroid occurs most frequently in patients who take minocycline for at least one year,^11^ but has been reported in those on the medication for durations ranging from 27 days to 20 years.^12^ Minocycline is also known to cause pigmentation of other tissues including the skin, bones, nails, mouth, and eyes.^11^ Cutaneous hyperpigmentation is also a known side effect of doxycycline administration in humans,^13^ but just one reported case of black thyroid in a person on doxycycline was located, where the black pigmentation was observed 12 days after starting doxycycline.^3^ Due to the paucity of data, there is no established mean administration duration of doxycycline associated with black thyroid development.

Reports of the antithyroid effects of minocycline in humans, such as the development of thyroiditis, raise concern for the clinical implications of black thyroid.^4^, ^14^ However, black thyroid is asymptomatic, and is generally considered to be a benign finding due to a lack of evidence demonstrating any correlation with thyroid disease.^5^, ^9^, ^11^ Attempts have been made to correlate black thyroid with an increased incidence of thyroid tumors in humans, particularly papillary thyroid cancer, but no causal relationship has been defined.^5^, ^8^, ^9^, ^17^ Black thyroid is generally an incidental surgical or autopsical finding due to the inability to appreciate it via imaging modalities, and the infrequency with which it is diagnosed on fine needle aspirate.^5^, ^9^, ^10^, ^12^, ^15^, ^16^ Therefore, a counter‐argument suggests that the reason black thyroid is found in patients with thyroid tumors is because tumor removal is a common reason to visualize the thyroid, allowing for discovery of the abnormal pigmentation.^5^, ^17^

Doxycycline and minocycline are chemically similar and have similar indications for use. Anecdotally, doxycycline is used much more commonly than minocycline in veterinary medicine. As previously stated, the vast majority of reports of black thyroid in humans are associated with chronic minocycline use,^1^ with one reported case associated with doxycycline.^3^ We suspect that black thyroid being reported once in an animal outside of an experimental setting^7^ could in part be due to the infrequency with which minocycline is prescribed. Other possible explanations include the limited number of diseases for which years long tetracycline administration is indicated, or physiologic variation resulting in a decreased proclivity for thyroid peroxidase reaction with tetracyclines as compared to humans. However, these hypotheses are speculative in nature.

Black thyroid is only rarely identified in human medicine, and the likelihood of its discovery can be assumed to be even less in veterinary medicine, where the pursuit of specialty care for thyroidectomy or the election of post‐mortem examination are done for only a small percentage of patients. This makes an inherently uncommon finding even less likely to be detected. In addition to infrequent minocycline use, these circumstances likely contribute to black thyroid being rarely reported in our veterinary patient population. The clinical implication of black thyroid has yet to be fully elucidated in human medicine. Due to the extreme rarity of black thyroid in animals, any ill effects of the condition, including any potential correlation with thyroid tumor formation, are currently unknown. At this time there is no evidence that performing a thyroidectomy is necessary or appropriate for black thyroid alone, regardless of patient species. Veterinary surgeons should be aware that dogs on long‐term doxycycline therapy may have this discoloration, so unnecessary total thyroidectomy can be prevented in affected animals.

## AUTHOR CONTRIBUTIONS

Stark SJ, DVM: Assisted in the surgery, performed a literature review, and drafted and revised the manuscript. Armstrong AR, DVM, PhD, DACVP: Reviewed histology samples, ordered special staining and IHC, drafted the histology section of the manuscript, revised the manuscript, and provided images for figures. Merickel JL, DVM: Was responsible for surgical management of the case and aided in manuscript revision. Gordon‐Evans WJ, DVM, PhD, DACVS, DACVSMR, DACVS (Small Animal): Oversaw the surgical management of the case and aided in manuscript revision. All authors provided a critical review of the manuscript and endorse the final version. All authors are aware of their respective contributions and have confidence in the integrity of all contributions.

## FUNDING INFORMATION

This report was not supported by any sponsor or funder.

## CONFLICT OF INTEREST STATEMENT

The authors have no conflicts of interest to disclose.

## Data Availability

Data sharing is not applicable to this article as no new datasets were generated or analyzed during the current report.
